# Immunoinformatics-aided rational design of a multi-epitope vaccine targeting feline infectious peritonitis virus

**DOI:** 10.3389/fvets.2023.1280273

**Published:** 2023-12-13

**Authors:** Mohit Chawla, Andrés Felipe Cuspoca, Nahid Akthar, Jorge Samuel Leon Magdaleno, Siriluk Rattanabunyong, Chonticha Suwattanasophon, Nathjanan Jongkon, Kiattawee Choowongkomon, Abdul Rajjak Shaikh, Tabarak Malik, Luigi Cavallo

**Affiliations:** ^1^Physical Sciences and Engineering Division, Kaust Catalysis Center, King Abdullah University of Science and Technology (KAUST), Thuwal, Saudi Arabia; ^2^Grupo de Investigación en Epidemiología Clínica de Colombia (GRECO), Universidad Pedagógica yTecnológica de Colombia, Tunja, Colombia; ^3^Centro de Atención e Investigación Médica–CAIMED, Chía, Colombia; ^4^Department of Research and Innovation, STEMskills Research and Education Lab Private Limited, Faridabad, Haryana, India; ^5^Department of Biochemistry, Faculty of Science, Kasetsart University, Bangkok, Thailand; ^6^Department of Social and Applied Science, College of Industrial Technology, King Mongkut’s University of Technology North Bangkok, Bangkok, Thailand; ^7^Department of Biomedical Sciences, Institute of Health, Jimma University, Jimma, Ethiopia

**Keywords:** feline coronavirus, feline infectious peritonitis, vaccine, immunoinformatics, reverse vaccinology, spike protein

## Abstract

Feline infectious peritonitis (FIP) is a grave and frequently lethal ailment instigated by feline coronavirus (FCoV) in wild and domestic feline species. The spike (S) protein of FCoV assumes a critical function in viral ingress and infection, thereby presenting a promising avenue for the development of a vaccine. In this investigation, an immunoinformatics approach was employed to ascertain immunogenic epitopes within the S-protein of FIP and formulate an innovative vaccine candidate. By subjecting the amino acid sequence of the FIP S-protein to computational scrutiny, MHC-I binding T-cell epitopes were predicted, which were subsequently evaluated for their antigenicity, toxicity, and allergenicity through *in silico* tools. Our analyses yielded the identification of 11 potential epitopes capable of provoking a robust immune response against FIPV. Additionally, molecular docking analysis demonstrated the ability of these epitopes to bind with feline MHC class I molecules. Through the utilization of suitable linkers, these epitopes, along with adjuvants, were integrated to design a multi-epitope vaccine candidate. Furthermore, the stability of the interaction between the vaccine candidate and feline Toll-like receptor 4 (TLR4) was established via molecular docking and molecular dynamics simulation analyses. This suggests good prospects for future experimental validation to ascertain the efficacy of our vaccine candidate in inducing a protective immune response against FIP.

## 1 Introduction

Feline coronavirus (FCoV) stands as the underlying agent responsible for feline infectious peritonitis (FIP), a widespread ailment afflicting both domestic and wild felids. This disease is particularly prevalent among cats dwelling in densely populated surroundings and displays global reach (1). The prevalence of FCoV hovers between 70 and 86%, with heightened susceptibility observed in kittens below the age of 2 years (2–4). Classified within the Coronaviridae family and the α-coronavirus genus, FCoVs are enveloped, positive-sense, single-stranded RNA viruses distinguished by their distinctively club-like spike proteins (5). Two discernible pathotypes of FCoV exist: feline enteric coronavirus (FECV) and feline infectious peritonitis virus (FIPV). While FECV is benign, markedly contagious, and often manifests asymptomatically, there are instances where it leads to mild diarrhea or even severe enteritis. Its mode of transmission primarily revolves around the fecal-oral route (6, 7). In contrast, FIPV can be lethal, does not transmit through the fecal-oral route, and arises from mutations (amino acid substitutions in spike proteins) in virulent FECV strains (6, 8). The mutations cause a cellular tropism switch of FIPV from gastrointestinal epithelium to monocytes/macrophages, which leads to the systemic infection of the virus (5). Among the cats infected with FCoV in multi-cat environments such as cat shelters, rescue centers, or breeding catteries, a small population (7–14%) are affected with FIPV (5). Cats suffering from FIP show clinical signs like weight loss, jaundice, anemia, lethargy, anorexia, fever, pale mucous membranes, lymph node enlargement, abdominal distension, dyspnea, and some neurological and ocular disorders (5). Furthermore, FIPV causes pyogranulomas, microcytosis, serous effusion in body cavities, phlebitis, lymphopenia, and serositis in the infected hosts (6, 8). Notably, FIP is responsible for 0.3–1.4% of feline deaths at veterinary clinics (5).

The genome of FCoV encompasses a repertoire of genetic elements, including 16 non-structural proteins (NSPs), five accessory proteins (3a, 3b, 3c, 7a, and 7b), and four structural proteins: spike, envelope, membrane, and nucleocapsid protein (8–10). The NSPs are necessary for the synthesis of viral RNA, whereas the envelope and membrane proteins help in viral assembly, maturation, and host cell interaction (10). The role of FCoV accessory proteins has not been well determined, but the accessory protein 3c has been associated with the intestinal replication of both FECV and FIPV (10, 11). The spike protein, a type I transmembrane protein, in coronavirus is vital for host cell type specificity (macrophage tropism), viral attachment, and fusion of the host’s cellular and viral membranes (8, 12). Furthermore, the spike protein is also important for the induction of cell-mediated immunity and antibody response in FCoV-infected felids (10). Moreover, the spike protein of FCoV is a significant determinant of virulence, pathogenesis, and the switch from a virulent FECV to a virulent FIPV (13). Previous studies have utilized a heptad repeat 2 peptide (57 amino acids) from the FECV spike protein to develop a recombinant oral vaccine candidate (13).

Currently, there is a lack of effective vaccines or clinically approved therapeutics for the treatment of feline coronavirus (FCoV) (14). FIP can be fatal to felids so it is important to develop vaccine candidates that could generate protective immunity from FcoV in cats.

Given the pivotal role of the FcoV spike protein in immune induction, both in terms of cell-mediated and humoral responses, as well as its involvement in FcoV pathogenesis and virulence, this study focuses on predicting epitopes within the spike protein for the development of a vaccine candidate against FIPV using immunoinformatics. The antigenic potential, allergenic attributes, and toxic properties of these epitopes were subjected to comprehensive evaluation employing diverse computational tools. Moreover, the scrutiny extended to molecular docking and molecular dynamics simulations, facilitating the analysis of interactions between the devised vaccine candidate and the immune cells specific to felines.

## 2 Materials and methods

### 2.1 Recovery and analysis of spike surface glycoprotein (S-protein) for FIPV

The amino acid sequence of the feline infectious peritonitis virus (FIPV) spike (S) protein, consisting of 1,452 amino acids, was obtained from UniProt (accession number: P10033). Subsequently, the antigenicity of the FIPV S-protein was assessed using Vaxijen 2.0, an online server (15).

### 2.2 Creating a vaccine contender: forecasting epitopes within S-protein via NetMHCpan 4.1

T-cell cytotoxic (Tc) epitopes derived from the feline infectious peritonitis virus (FIPV) spike (S) protein were predicted utilizing the NetMHCpan 4.1 web server (16). The FIPV S-protein sequence was employed as an input in FASTA format within the NetMHCpan 4.1 web server, and epitopes with a length of 9 amino acids (9-mers) were predicted using default parameters. As the S-protein of FIPV shares 100% sequence identity with the spike protein of Canine Coronavirus, the DLA alleles (DLA-8803401, DLA-8850101, DLA-8850801) were selected for epitope prediction. The Tc epitopes exhibiting strong binding affinity were subsequently selected for further analyses.

### 2.3 Epitope elucidation from S-protein for FIPV and molecular docking of identified epitopes with feline MHC-I

The epitope’s antigenicity, allergen potential, and toxicity were predicted using Vaxijen 2.0, AllergenFP, and ToxinPred webservers, respectively (15, 17, 18). The default parameters were employed while utilizing these web servers, and the epitope sequences were entered in single-letter code format. For Vaxijen 2.0, the target organism was set as the virus. Epitopes that were predicted to possess antigenic properties, be non-allergenic, and be non-toxic were chosen for inclusion in the final vaccine design.

The screened peptides were modeled using the AmberTools program (19). Subsequently, molecular screening of these peptides was conducted using the AutoDock Vina software (20). During the screening process, the peptides were allowed to exhibit flexibility, while the protein remained in a fixed conformation. For the study, the chain A of the PDB ID: 5XMF (21) was chosen, which represents the crystal structure of feline major histocompatibility complex (MHC) class I. The binding site was defined using the co-crystalized gag protein. The grid used for docking calculations was positioned at coordinates (32, 22, 21.5) with a size of 30 Å in each dimension (X, Y, and Z). The peptide demonstrating the most negative binding energy was considered to possess the highest binding affinity.

### 2.4 Designing the final vaccine construct

In the vaccine formulation, we incorporated the non-toxic region of cholera toxin (CtxB), which exhibits enhanced affinity for the “prototype” ganglioside (GM1) when positioned at the N-terminal of the vaccine (22). GM1 is manifested on the cell surface of specialized antigen-presenting cells (APCs) like dendritic cells and B-cells. This presence triggers an increase in the expression of major histocompatibility complex class II (MHC-II) and fosters differentiation in immunoglobulins (Ig) (23). This results in improved uptake of antigens, thereby enhancing antigen availability and facilitating better interaction with TCD4 cells. To further enhance this immune stimulation, we included a combined epitope of tetanus and diphtheria toxoid (TpD), which has been identified as a universal adjuvant for TCD4 cells, potentially surpassing the efficacy of “PADRE,” a peptide known to promiscuously bind to several Human Leukocyte Antigen-DR (HLA-DR) molecules (24). *In vitro* studies have demonstrated the ability of TpD to induce the production of neutralizing antibodies (25) and confer protection on mucous membranes (26). These outcomes persist across different mammalian species and are marked by the creation of enduring CD4^+^ T central memory cells, the synthesis of neutralizing antibodies, and the release of Interferon-gamma (IFN-γ) and TNF-α cytokines. This pattern signifies a T-helper cell (Th1)-dominant immune response (27). Toward the C-terminus of the vaccine, we incorporated the final subunit of the *Escherichia coli* type 1 fimbria (FimH), which has been found to interact with Toll-like receptor 4 (TLR4) in a dependent manner. This interaction promotes the maturation, activation, and proliferation of local dendritic cells and peripheral cells and exhibits a more favorable and safe regulation of MHC class I and class II molecules compared to lipopolysaccharide (LPS). Similar to TpD, FimH also stimulates the production of IFN-γ and TNF-α (28, 29). Notably, the use of FimH has been recognized for its efficacy in mucosal immunity (28).

To elicit a targeted cellular and humoral response while minimizing the interaction between the input protein and the host, we utilized a specific fragment of the Spike protein known for its interaction with host cells. This fragment was employed to induce a cell-adjuvanted response involving both T cells and B cells. Previous studies have demonstrated the utility of utilizing fragments of the target protein that bind to the host receptor in vaccine design, resulting in the generation of neutralizing antibodies and a robust cellular response (30). Appropriate linkers, such as “GGGGS” and “EAAAK,” were incorporated to form rigid or flexible protein configurations based on the desired biological activity. Increased flexibility allows for greater variation in distance between the N and C termini of the fusion protein, impacting antigenic presentation by effectively separating embedded domains of interest (31, 32). The inclusion of lysine (K) in vaccine constructs enhances solubility by avoiding B cell epitopes and offers improved immune response, while also providing a favorable cleavage site for proteases within lysosomes, an essential step in CD4 T cell presentation (33, 34).

### 2.5 Molecular modeling and docking study

The three-dimensional (3D) structure prediction of the FIPV multi-epitope vaccine (FIPV-MEV) was conducted using AlphaFold v2 (35). However, the structure of *Felis catus* Toll-like receptor 4 (TLR4) (UniProt ID: P58727) was directly obtained from the AlphaFold database (36). In the case of the TLR4 structure, only the extracellular domain spanning amino acids 24–632 was retained, while other regions were excluded. The predicted local distance difference test (pLDDT) scores were accessible through Alphafoldv2.0, while the Ramachandran plots and Z-scores were produced (37) employing the ProCheck and ProSA webserver to appraise the structural quality (38). For the docking of FIPV-MEV with TLR4, the HADDOCK server (39) was engaged, adhering to the default settings. It is important to note that the sequence of FimH, integrated into the FIPV-MEV structure (encompassing residues 586–610) and acknowledged for its capability to activate TLR4 (28, 40), was distinctly marked as the “Active residues” within the FIPV-MEV architecture. Conversely, for TLR4, the core domain encompassing the human LRR8 and LRR9 segments (residues 225–245) was specified as the “Active residues” during the implementation of the HADDOCK program (41).

### 2.6 Molecular dynamics simulations study

The Molecular Dynamics (MD) simulations were conducted utilizing the GROMACS 2022 simulation software (42). The cubic simulation box contained the multi-epitope vaccine complexed with TLR-4, immersed in a solvating environment consisting of a combination of atomistic TIP3P and coarse-grain WatFour (WT4) water models (43). Coarse-grain ions NaW and ClW were included. The protein parameters, TIP3P water model, and SIRAH force field (44) were used for the WT4 coarse-grain water model and ions, while the CHARMM-36 (45) force field was applied for the proteins. To ensure overall neutrality and to replicate physiological conditions, Na + and Cl- ions were added, resulting in a bulk ionic strength of 0.15 M. The simulation box was composed of 1,048 NaW, 1,068 ClW, 23,990 TIP3P water molecules, and 51,000 WT4 water molecules, totaling 142,215 atoms. A two-step minimization approach was adopted: first, a steepest descent minimization with 500,000 steps, followed by a 50,000-step conjugate gradient minimization. Equilibration of the system was accomplished with 100 ps of NVT equilibration and 100 ps of NPT equilibration, both performed without restraints. Subsequent production simulations spanned 50 ns, employing the NPT ensemble. The temperature was maintained at 310 K (physiological temperature) using a velocity rescaling approach with a coupling time of 0.1 ps. The pressure was controlled at 1 atm during NPT simulations using the Parrinello-Rahman barostat (46) with a coupling time of 2 ps. Integration of equations of motion was carried out using the leapfrog algorithm with a time step of 2.0 fs. Electrostatic interactions were computed using the particle mesh Ewald (PME) summation method (47), with Coulomb and van der Waals cut-offs set at 1.0 nm. Periodic boundary conditions were applied along the x, y, and z axes to emulate bulk behavior. Bond lengths involving hydrogen were constrained using the LINCS algorithm (48). The simulation coordinates were recorded in trajectory files every 20 ps. GROMACS tools were employed for trajectory processing and various analyses. Visualization and molecular graphics were generated using PyMOL software (49), while complex illustrations were prepared with VMD (50). For generating the protein–protein interaction map, ProLIF (51) was utilized. Plots were generated using Matplotlib (52).

## 3 Results

### 3.1 Retrieval and analysis of FIPV S-protein sequence

The FIPV S-protein was identified by the UniProt ID P10033 and consists of 1,452 amino acids and exhibits a 4/5 annotation score. It is associated with the Feline Coronavirus strain FIPV WSU-79/1146. The potential antigenicity of the FIPV Spike protein was assessed by utilizing the Vaxijen web server, which resulted in a Vaxijen score of 0.5234. This score substantiates the protein’s antigenic characteristics.

### 3.2 Epitope prediction from S-protein for vaccine candidate design and their molecular docking with FLA-E*01801 protein

**Figure 1:**
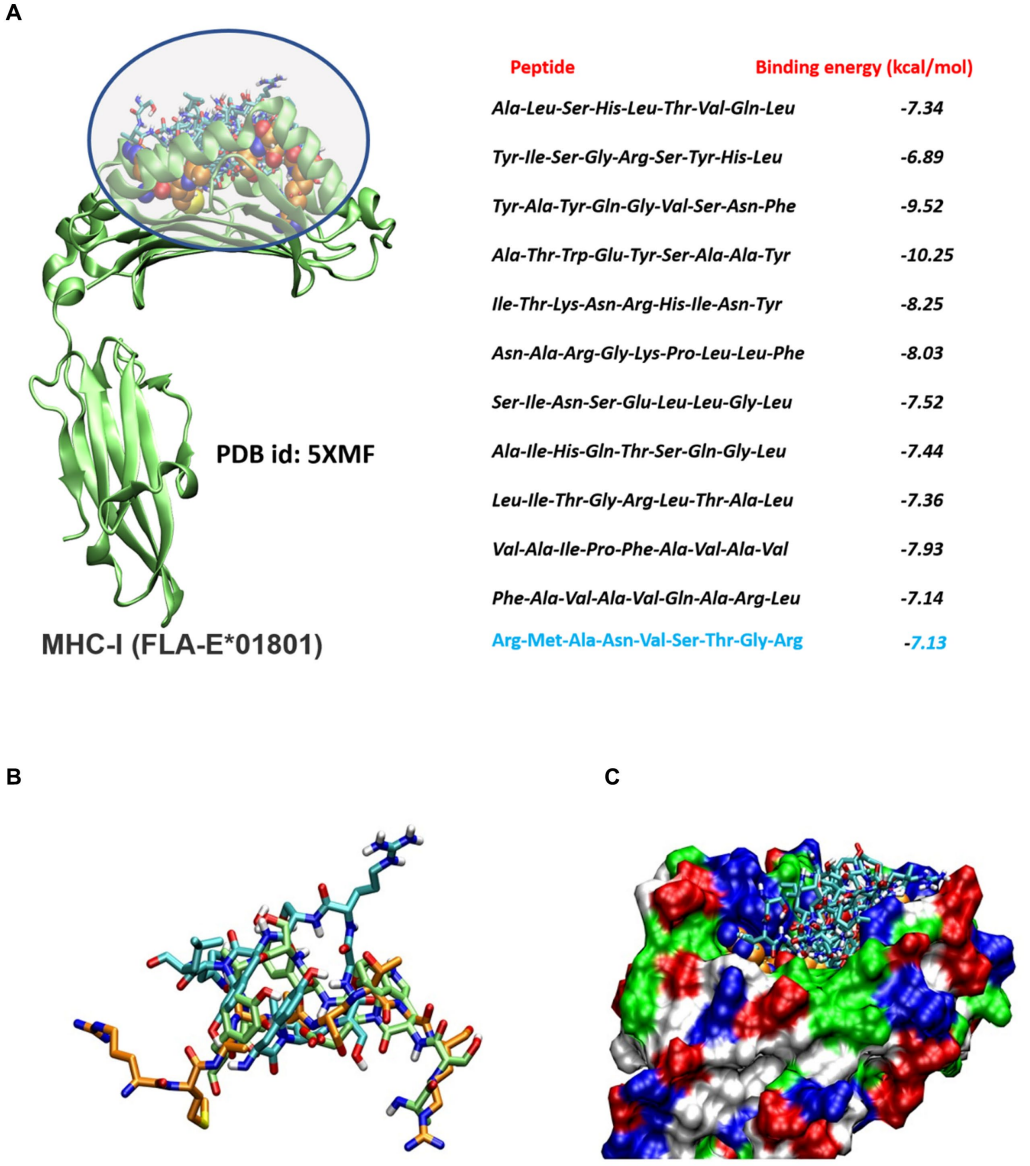
**(A)** Molecular docking analysis of the screened peptides and FLA-E*01801 major histocompatibility complex class I (MHC-I). Peptide binding site within the FLA-E*01801 MHC-I structure, depicted by highlighting the region. The screened peptides are represented as stick models. The reference peptide (gag-peptide) co-crystallized with the FLA antigen is shown as a spherical model. The right panel displays the corresponding binding affinity values, with the binding affinity of the reference gag peptide highlighted in blue color. **(B)** Molecular docking analysis of the screened peptides and FLA-E*01801 major histocompatibility complex class I (MHC-I). The screened peptides compound 2 (cyan), compound 4 (lime), and the gag-protein (orange) are represented as stick models. **(C)** Protein is shown as a molecular surface and all docked conformation is shown as a stick model.

A comprehensive number of 4,332 Tc epitopes were forecasted from the FIPV-S protein employing the MetMHCpan 4.1 webserver. Among these predicted epitopes, 89 were identified as strong binders and selected for subsequent analysis (Supplementary Table S1). After careful evaluation, 11 Tc epitopes were chosen for vaccine design based on their predicted antigenicity, non-allergenicity, and non-toxicity (Table 1). Additionally, molecular docking analysis was conducted on the 11 screened peptides with FLA-E*01801-MHC-I, which resulted in the binding of these peptides to the same binding sites as the reference co-crystallized gag-peptide in FLA-E*01801 protein, see Figure 1. The binding affinity values for all the peptides examined were comparable to the reference gag-peptide bound to the FLA-E*01801 protein, indicating a strong affinity of the screened peptides for the FLA-E*01801 protein.

**Table 1:** Final epitopes selected for vaccine design and their properties.

Epitope type	Protein ID	Peptide	Binding affinity (nM)	Vaxijen score	Antigen/Non-antigen	Allergenicity	Toxicity
T_c_ cell	P10033	ALSHLTVQL	202.74	0.8919	Antigen	Non-allergen	Non-toxin
		YISGRSYHL	46.98	0.8704	Antigen	Non-allergen	Non-toxin
		YAYQGVSNF	622.48	0.5752	Antigen	Non-allergen	Non-toxin
		ATWEYSAAY	261.81	0.4778	Antigen	Non-allergen	Non-toxin
		ITKNRHINY	2282.82	1.3569	Antigen	Non-allergen	Non-toxin
		NARGKPLLF	3583.25	1.1829	Antigen	Non-allergen	Non-toxin
		SINSELLGL	136.93	0.6903	Antigen	Non-allergen	Non-toxin
		AIHQTSQGL	965.25	0.7751	Antigen	Non-allergen	Non-toxin
		LITGRLTAL	339.95	0.9134	Antigen	Non-allergen	Non-toxin
		VAIPFAVAV	577.13	0.8730	Antigen	Non-allergen	Non-toxin
		FAVAVQARL	345.46	1.1472	Antigen	Non-allergen	Non-toxin

### 3.3 Design of final FIPV vaccine construct

**Figure 2:**
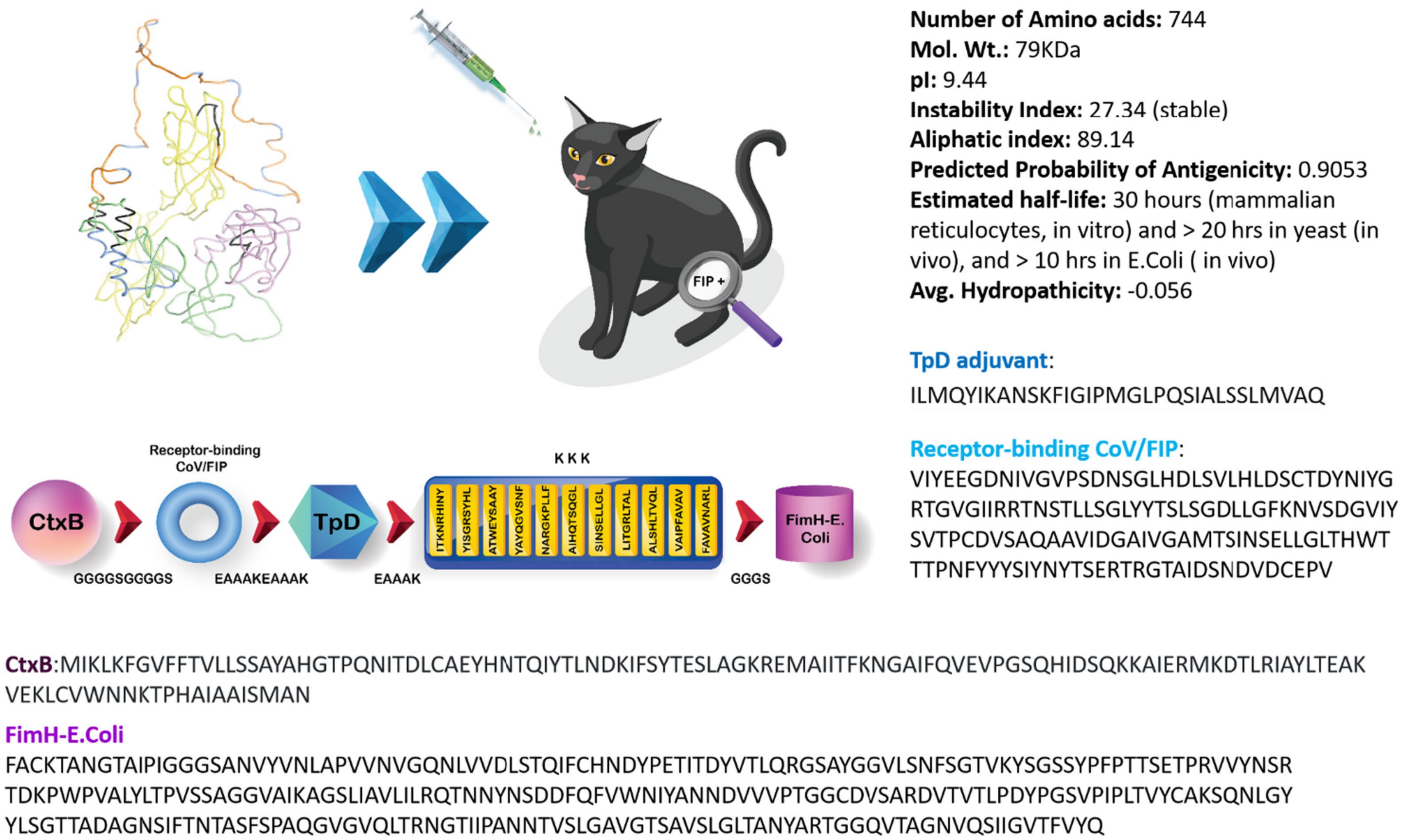
Illustration outlining the schematic design of the projected FIPV-MEV construct, accompanied by the corresponding amino acid sequences for the employed constructs, along with an overview of the physicochemical attributes of the anticipated FIPV-MEV.

In summary, a multi-epitope peptide-based vaccine construct targeting Tc epitopes, a receptor-binding domain, and relevant adjuvants (as listed below) was designed by linking 11 epitopes together using various linkers. The goal was to create a stable, antigenic, and non-allergenic vaccine construct specifically for FIPV. The resulting vaccine construct consists of a total of 744 amino acids, and its amino acid sequence is shown in Figure 2. Furthermore, Figure 2 presents the physicochemical attributes of the ultimate vaccine construct, encompassing characteristics such as the isoelectric point, atom count, aliphatic index, and other pertinent properties.

### 3.4 Stimulation of the immune system by vaccine construct components

**Figure 3:**
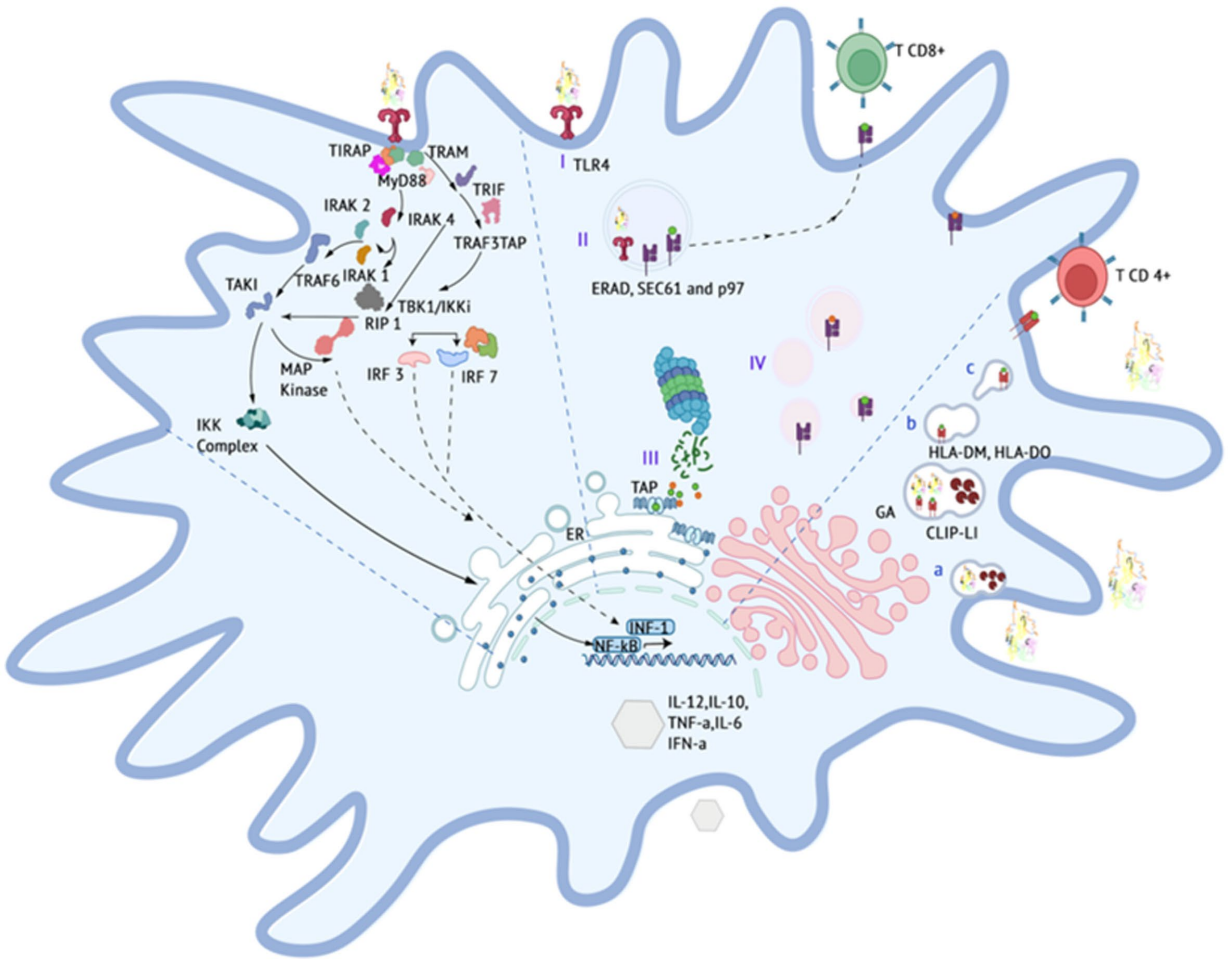
Expected events occuring in the immune system, particularly the stimulation of the immune system by vaccine construct components.

The proposed vaccine construct demonstrates a specific affinity for the TLR4 receptor through its interaction with the fimbrial end (FimH) of the *E. coli* type 1 fimbria. This interaction is specifically targeted toward TLR4 receptors present in dendritic cells derived from bone marrow, where they are moderately expressed in felines (53). Upon binding, it initiates a canonical interaction that triggers various cellular processes, including maturation, transcription, autophagy, endocytosis, phagocytosis, and oxidative bursts (54, 55). The signaling pathway following the binding of the FIPV-MEV construct to the TLR4 receptor possibly involves cytoplasmic dimerization of the Toll IL-1 receptor (TIR), see Figure 3. This dimerization facilitates the formation of a new assembly site, allowing the recruitment of adapter proteins that initiate two distinct intracellular signaling mechanisms (56, 57).

The initial mechanism entails the interaction between Toll/Interleukin-1 receptor domain-containing adapter protein (TIRAP), which then recruits myeloid differentiation primary response protein 88 (MyD88). This recruitment triggers the activation of interleukin-1 receptor-associated kinase (IRAK) proteins, subsequently initiating the activation cascade of tumor necrosis factor receptor (TNFR)-associated factor 6 (TRAF6) (58). The pathway progresses with the participation of the transforming growth factor (TAK1) and the NF-kappa-B kinase inhibitor complex (IKK), culminating in the translocation of NFkB subunits to the cell nucleus. Consequently, this translocation prompts the transcriptional upregulation of proinflammatory cytokines and chemokines, notably including tumor necrosis factor (59).

The second mechanism, distinct from MyD88 involvement, centers on the TRIF-related adapter molecule (TRAM), which instigates the activation of TIR domain-containing adapter-inducing interferon (TRIF). This activation, in turn, triggers receptor-interacting protein (RIP1) and TRAF6 activation, thereby initiating the activation cascade of NFkB and MAPK pathways. Additionally, TRIF interacts with TRAF3, leading to the activation of TRAF family member-associated NF-kappa-B activator binding kinase (TBK1) / I-kappa-B kinase i (IKKi). This activation, subsequently, activates interferon regulatory factors (IRF3) and (IRF7). These factors undergo nuclear translocation and facilitate the transcription of type 1 interferons (Type 1 IFNs).

Collectively, these pathways establish a pro-inflammatory innate immune response, leading to the production of cytokines and chemokines, upregulation of molecules involved in the adaptive immune response, activation of T cells toward an effector phenotype, and co-stimulation of other antigen-presenting cells (55).

TLR-4 is capable of internalizing the FIPV-MEV construct, facilitating cross-presentation and stimulation of TCD-8 cells. This process begins with the engulfment of the construct in the phagosome (I) and subsequent denaturation through pH-dependent mechanisms or transport to the cytoplasm via degradation machinery associated with the endoplasmic reticulum (ERAD), such as SEC61 or the hexameric AAA (ATPase) p97 (60). Proteasomal cleavage generates multiple peptide fragments (II), which are then transported to the ER through transporter proteins (TAPs) and assembled with MHC class I molecules. The assembled complex undergoes trafficking from the Golgi apparatus (III) to the cell membrane (61).

The proposed scheme includes the possibility of MHC-II presentation, which takes place within lysosomal compartments. In this context, the denaturation of the vaccine construct within the lysosomal environment results in the generation of short peptides under conditions of low pH and reducing agents. These conditions are conducive to the appropriate maturation of lysosomal proteases (a). The formation of a stable immune complex between the short peptides and MHC-II requires the removal of the invariant chain (LI) protein and its CLIP fragment from the native MHC-II framework. Molecule counterparts in felines, such as human leukocyte antigen DM (HLA-DM) and human leukocyte histocompatibility complex DO (HLA-DO), participate in the stabilization and localization of the peptides. Ultimately, this complex migrates to the plasma membrane, where it activates CD4 T cells.

In terms of B cell stimulation, the vaccine construct incorporates a host binding prediction site to enhance its interaction with B cells. This interaction aims to induce the generation of neutralizing antibodies that can prevent the internalization of the virus.

### 3.5 Modeling and docking of TLR4 and FIPV-MEV construct

Given the unavailability of experimental TLR4 structures for *Felis catus*, the TLR4 structure (Uniprot ID: P58727) was sourced from the AlphaFold database. The obtained structure displayed robust pLDDT values, notably exceeding 90 for most residues, reflecting the high-confidence nature of the prediction (Supplementary Figure S1). Subsequently, the 3D structure of the FIPV-MEV construct was projected using Alphafoldv2.0, yielding elevated pLDDT values above 90 for regions pertinent to the vaccine construct, including the sections where the FimH and the receptor binding to the host sequence were inserted. Conversely, the regions housing adjuvants/linkers and epitope sequences (amino acids 309–461) exhibited lower pLDDT values under 50, implying a structurally disordered state for this segment (Supplementary Figure S1).

**Figure 4:**
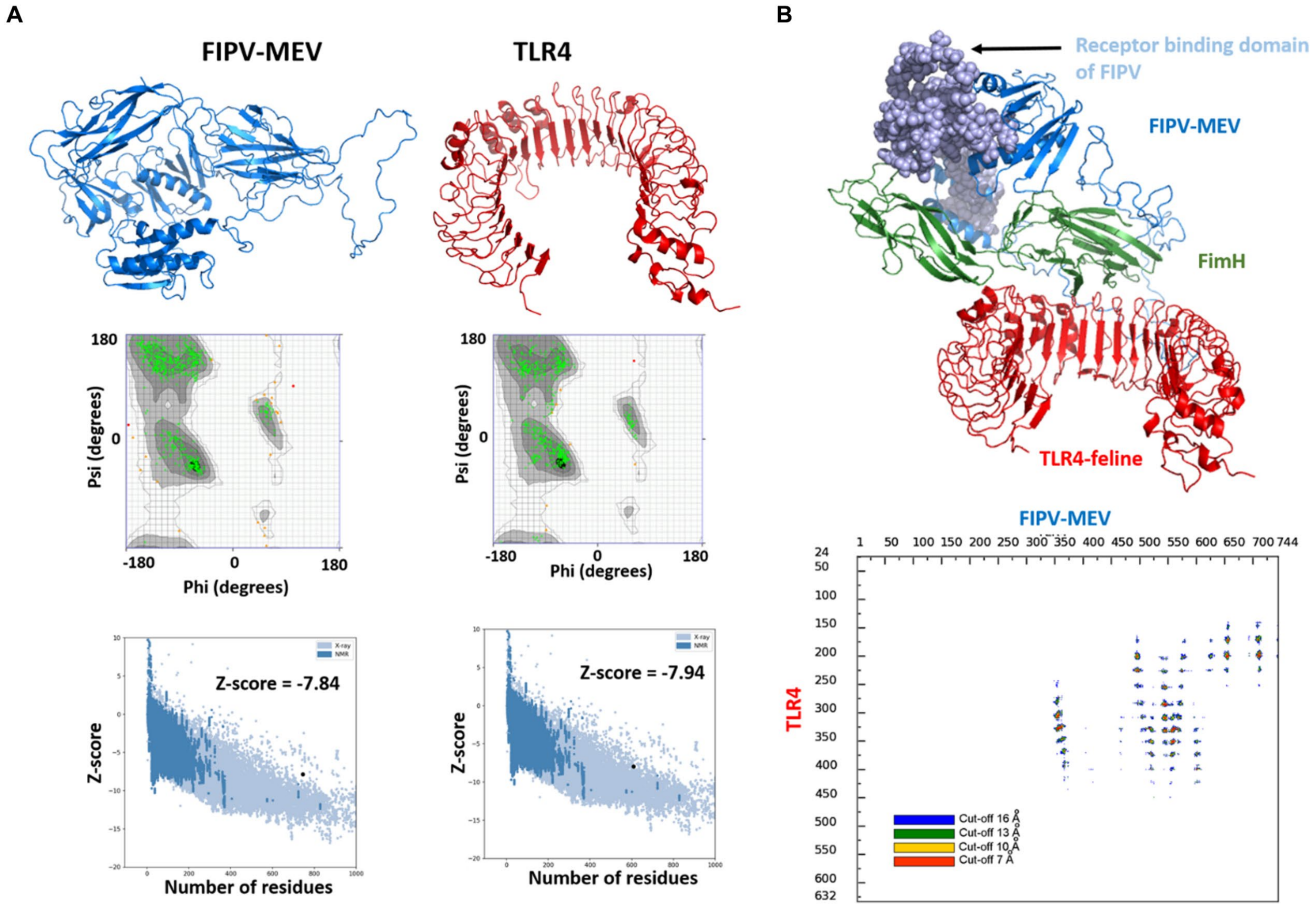
Modeled 3D structures of **(A)** FIPV-MEV construct and TLR4 and respective Ramachandran plots and Z-scores calculated by Pro-SA webserver. **(B)** Molecular docking between FIPV-MEV and TLR4 with docked complex (vaccine construct colored with different components, and TLR4 receptor in red). Interaction map of TLR4 and FIPV-MEV construct with residues that are interacting with a given threshold distance are presented in different colors.

To further validate the structural quality, Ramachandran plots and Z-scores from the ProSA webserver were employed. For the TLR4 receptor, approximately 81.6% of amino acids resided within the core acceptable region. Approximately 18% occupied the allowed and generously allowed regions, while only 0.4% fell into the disallowed region (Figure 4A). Regarding the FIPV-MEV construct, 66.9% of residues were positioned within favored regions, and 24.5% of residues were situated within allowed and generously allowed regions. However, 8.6% of residues were situated in disallowed regions (Supplementary Figure S2). Since a high proportion of residues fall under the disallowed region, we performed refining of the FIPV-MEV model by carrying out 50 ns molecular dynamics simulations for the FIPV-MEV construct and recalculated the Ramachandran plot. Our results showed a significant improvement in the proportion of residues in favored regions and only a small percentage (0.3%) in the disallowed regions, see Figure 4A, indicating a refined model. Structural superimposition of the FIPV-MEV construct before and after the simulation run is illustrated in Supplementary Figure S2. The refined FIPV-MEV model was used for further docking studies.

To delve into the interaction mechanism between the MEV construct and TLR4, which is pivotal in orchestrating immune responses, we conducted molecular docking between FIPV-MEV and TLR4 utilizing the HADDOCK 2.4 web server. Default parameters were employed for this docking process; additional particulars are outlined in the Methods section. The final structure of the FIPV-MEV and TLR4 complex was selected from the top-ranked cluster with the lowest HADDOCK score. This complex structure, depicting the molecular docking outcome, is depicted in Figure 4B. Moreover, to gain more insight, we generated “distance range maps” for the docked complex using the COCOMAPS tool (Figure 4B). Contact instances were determined by considering a cutoff distance of 5 Å between two atoms. Specifically, we identified 79 contacts between hydrophilic residues, 39 contacts involving hydrophilic and hydrophobic residues, and an additional 14 contacts among two hydrophobic residues.

### 3.6 Vaccine construct-TLR4 receptor complex stability

**Figure 5:**
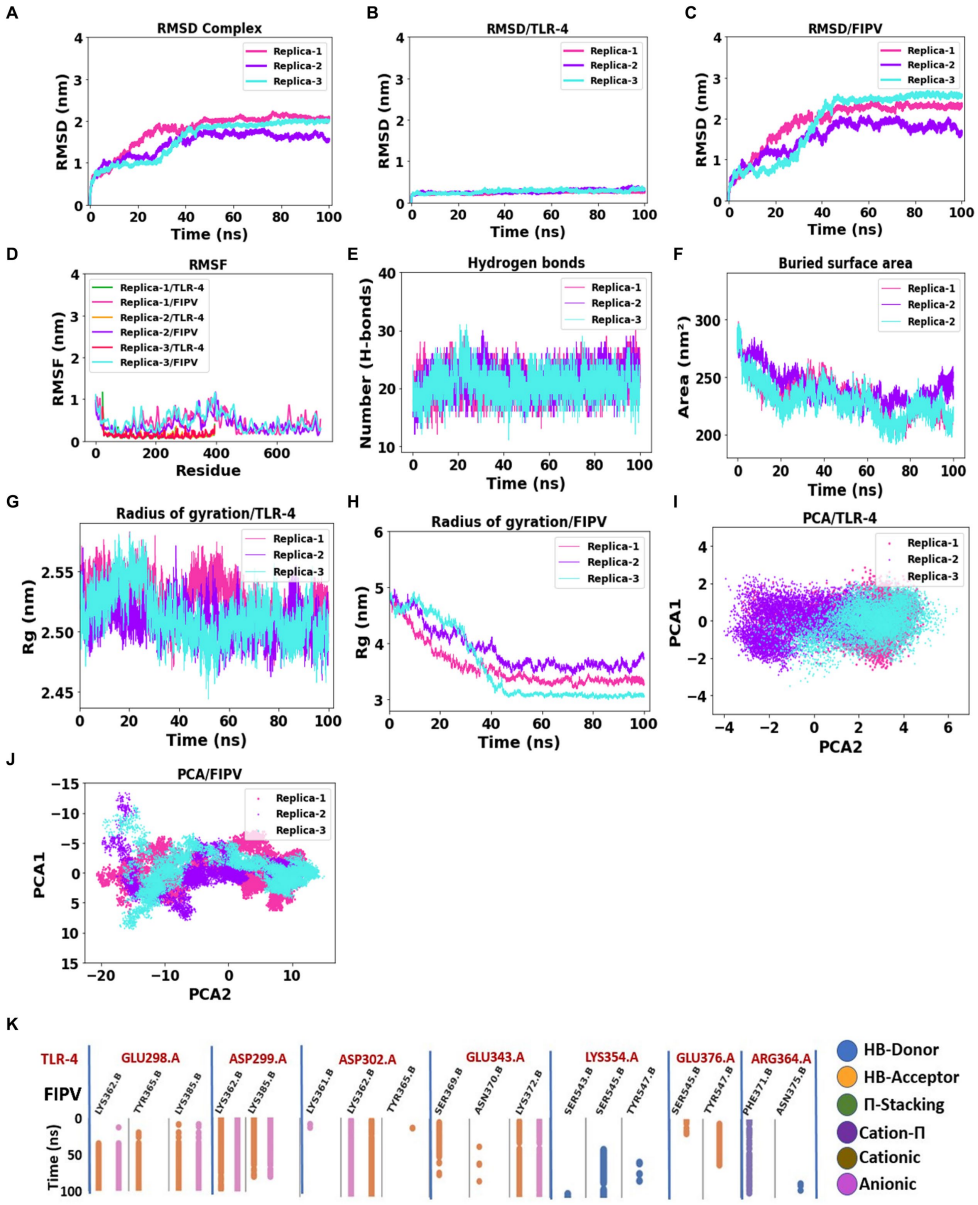
Assessment of complex stability: FIPV-MEV construct bound with TLR4 receptor. **(A–C)** Temporal progression of the backbone RMSD for the entire complex, FIPV-MEV, and TLR4 individually, over the MD simulation period. **(D)** Diagrams portraying the backbone RMSF fluctuations. **(E)** Evolution of hydrogen bond numbers between TLR4 and FIPV-MEV construct throughout the simulation. **(F)** Altered buried surface area trends across the MD simulations for the three replicates. **(G,H)** Fluctuations in the radius of gyration for TLR4 and FIPV-MEV. **(I,J)** Principal component analysis outcomes elucidating TLR4 and FIPV-MEV behavior. **(K)** Fingerprint analysis providing insights into the enduring stability of intermolecular interactions within the FIPV-MEV and TLR4 network across the simulation duration.

Molecular dynamics simulation has emerged as a valuable method for investigating the stability and analysis of biological systems (62–67). In this study, we employed the GROMACS software to evaluate the stability of a multi-epitope vaccine complexed with TLR-4. Three independent molecular dynamics simulations, each lasting 100 ns, were conducted for the FIPV-MEV complexed with TLR4 using different initial velocities, yielding highly comparable results. Figure 5 presents the calculated parameters for all three replicates, demonstrating consistent outcomes. To maintain clarity, we will present the outcomes solely for the initial replicate.

To evaluate the stability and possible conformational changes within the complex, we calculated the root mean square deviation (RMSD) of the backbone from its initial configuration. Notably, the complex exhibited a substantial RMSD, with an average measurement of 1.78 ± 0.42 nm (Figure 5A). Notably, the complex reached a stable state after approximately 40 ns of simulation, as depicted in Figure 5A, and maintained stability throughout the entire simulation duration across all three replicates. The TLR-4 receptor exhibited remarkable stability, with an average RMSD value of 0.24 ± 0.02 nm (Figure 5B). Conversely, the multi-epitope vaccine construct, characterized by long loops accommodating epitopes and linkers, displayed considerable flexibility, resulting in an average RMSD of 1.94 ± 0.54 nm (Figure 5C).

Subsequent exploration involved the examination of root mean square fluctuation (RMSF) individually for both TLR-4 and the vaccine construct. Notably, the RMSF analysis unveiled specific residues, primarily located in peripheral regions, which exhibited pronounced fluctuations, registering an average measurement of 0.48 ± 0.30 nm (Figure 5D). In stark contrast, TLR-4 demonstrated limited flexibility, evidenced by its notably lower average RMSF values of 0.13 ± 0.08 nm (Figure 5D).

Furthermore, we scrutinized the number of intermolecular hydrogen bonds within the MEV-TLR4 complex over the course of 20 ns, revealing a consistent count throughout the simulation period (Figure 5E). Additionally, the buried surface area at the interaction interface of the vaccine construct and TLR-4 maintained stability across all three replicates throughout the simulation duration (Figure 5F). These comprehensive analyses collectively underscore the robustness and consistency of the interface interactions between TLR-4 and the vaccine construct.

The gyration analysis demonstrated that both the FIPV protein and TLR-4 receptor maintained their compactness and shape during the simulation, highlighting the balanced interactions and forces governing their structures, thereby preventing significant conformational changes or unfolding, see Figures 5G,H.

To investigate significant motions during the simulation of MEV and TLR-4, principal component analysis (PCA) was employed. Figures 5I,J displays the conformational samplings of the MEV-TLR-4 replicas in the essential subspace, illustrating the global motions along PC1 and PC2 projected by the Cα atom. All complexes exhibited stability and occupied a confined phase space in the two-dimensional projection, indicative of a stable complex across all three replicates. The 2D projection of PCA revealed small conformational subspaces and similar patterns of motion, further confirming the formation of a stable complex in all three replica analyses.

Figure 5K illustrates the molecular interactions within the interaction interface between MEV and TLR-4, as observed in the molecular dynamics simulation results. To focus on relevant interactions, a cutoff threshold was applied based on the occupancy of interactions throughout the 100 ns simulation. The chosen threshold of 0.3 indicates that these interactions were present for at least 30% of the entire simulation duration. Notably, the side chains of TLR-4 involved in these interactions are as follows: Glu298, which forms hydrogen bonds and anionic interactions with the side chains of Lys362, Tyr365, and Lys385 of MEV; Asp299, which establishes hydrogen bond and anionic interactions with the side chains of Lys362 and Lys385. Additionally, TLR-4’s Asp302 mainly exhibits hydrogen and anionic interactions with Lys362. Moreover, the TLR-4 Glu343 forms interactions as a hydrogen acceptor bond and anionic interactions with Lys372 of MEV. Conversely, the side chain of TLR-4 Lys354 forms an H-bond donor interaction with the Ser545 side chain of MEV, and TLR-4’s Arg364 forms a Cation-π interaction with Phe371 of MEV. This fingerprint analysis highlights the significance of these interactions with high occupancy throughout the entire molecular dynamics simulation, emphasizing their role in promoting stability within the FIPV-MEV and TLR4 interface.

**Figure 6:**
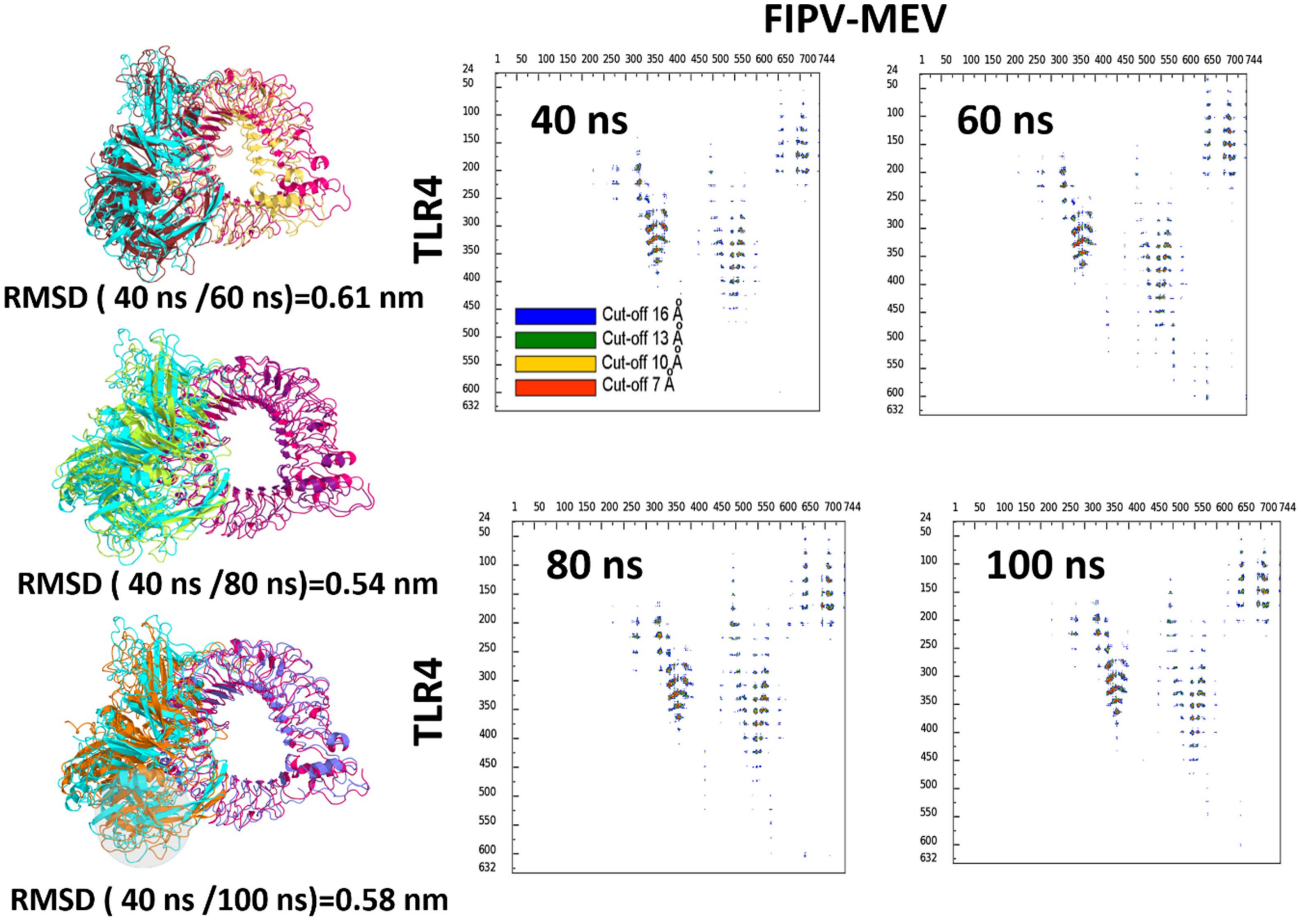
Superimposition of selected snapshots of the TLR4 and the FIPV-MEV construct and their respective RMSD values for the first replicate simulations. Contact Maps Illustrating Interactions: Depiction of intermolecular contacts based on distance for identical snapshots. The dots at the juncture of two residues are color-coded red, yellow, green, and blue, indicating proximity of any atom pair within 7, 10, 13, and 16 Å, respectively.

The robustness of the structural integrity was reaffirmed through the superimposition of the complete complex involving the vaccine construct and the TLR-4 receptor. This analysis showcased a favorable alignment between the structures, as depicted in Figure 6. The high root mean square deviation (RMSD) values observed in the structures depicted in Figure 6 can be attributed to the presence of unaligned flexible regions within the vaccine construct, particularly the long and flexible loops. Notwithstanding these structural discrepancies, the interaction pattern among the residues engaged in the interaction between FIPV-MEV and TLR-4 exhibited steadfast consistency. To delve deeper into the interface connecting TLR-4 and the docked vaccine construct, specific snapshots were subjected to interface analysis using the COCOMAPS tool (68) (Figure 6). This analysis generated contact maps, offering a visual representation of pairwise distances between residues belonging to the vaccine construct and TLR-4. Within these contact maps, dots are color-coded in red, yellow, green, and blue, denoting distances below 7, 10, 13, and 16 Å, respectively. Figure 6 notably demonstrates the interface’s unwavering stability across the sampled snapshots, as evidenced by the inter-residue contacts.

## 4 Discussion

Depending on the biotypes, FCoVs can either cause benign or systemic and lethal infections in both domestic and wild felids (1, 6). There is a lack of an effective vaccine and clinically approved drugs for the treatment of FCoV (14). However, different studies have identified potential antiviral drugs that could be effective for FIP treatment (69–72). For instance, an *in vitro* study demonstrated that ERDRP-0519, a non-nucleoside inhibitor targeting RNA polymerase, effectively inhibits FIPV replication in a dose-dependent manner (69). Similarly, diphyllin, a vacuolar ATPase blocker, and its nano formulation inhibited FIPV replication (71). Nelfinavir, an HIV-1 protease inhibitor, in combination with *Galanthus nivalis* agglutinin, has shown a synergistic effect in inhibiting FCoV replication (73). Another study screened 90 compounds and identified anti-FIPV activity in 26 different compounds (74). Moreover, recombinant feline interferons, omega-a and omega-b, have also shown antiviral activity against FCoV (75).

Other than the antiviral compounds, numerous efforts have been made to develop vaccines against FCOV (13, 76, 77). Oral administration of a recombinant vaccine based on the *Bacillus subtilis* surface display technique expressing FECV heptad repeat 2 domain peptide protected against FECV infection and ameliorated digestive tract pathology in mice models (13). A live attenuated FIP vaccine made using the deletion of ORF 3abc protected cats from lethal FIPV challenge (78). Vaccination with recombinant baculovirus-expressed nucleocapsid protein of FIPV did not induce virus-neutralizing antibodies but increased the survival rate of cats challenged with heterologous FIPV in comparison to the control group (79). However, a DNA vaccine entailing plasmids encoding FIPV nucleocapsid and membrane failed to protect kittens from FIP (80).

In the absence of effective therapies against FIP, it is imperative to research novel, safe, and effective strategies, such as the development of vaccines, immunotherapies, and antiviral drugs, to treat and protect cats from FIV infections. One possible avenue is immunoinformatics, which could be an inexpensive and rapid method of designing new vaccine candidates for the protection of felids from fatal FIPV infection. This is the first study aiming to develop a multi-epitope vaccine candidate against FIPV using immunoinformatics. However, in an earlier study, a peptide-based vaccine consisting of two T-helper-1 cell epitopes (GQRKELPERWFFYFLGTGPH and EPLRFDGKIPPQFQLEVNRS) derived from nucleocapsid protein of FIPV in conjugation with feline CpG-oligodeoxynucleotides adjuvant prevented cats from contracting FIPV (81). A similar study also identified two epitopes, NNYLTFNKFCLSLSPVGANC (from spike protein) and QYGRPQFSWLVYGIKMLIMW (from membrane protein), of FIPV that induced T-helper 1 activity in specific pathogen-free cats when administered along with feline CpG-oligodeoxynucleotides adjuvant (82). Previously, an immunoinformatics approach has been used for the development of vaccine candidates against various animal viruses, such as canine circovirus, avian influenza virus, lumpy skin disease virus, and African swine fever virus (66, 83–86).

In the present study, we utilized computational methods to predict 11 antigenic epitopes derived from the S-protein of FIPV. These epitopes were found to be non-toxic, non-allergic, and capable of interacting with the feline MHC-I molecule. To design an effective FIPV vaccine, these epitopes were combined with a receptor binding domain of FIPV and relevant adjuvants (CtxB, TpD, and FimH) using different linkers. The resultant vaccine construct, comprising 744 amino acids, demonstrates robust stability, antigenicity, and non-allergenic attributes. To deepen our understanding of the interaction between the vaccine contender and feline Toll-like receptor 4 (TLR4), we conducted molecular docking and molecular dynamics simulations, effectively affirming the steadfastness of this interaction. These computational evaluations provide valuable corroboration for the immunogenic potential of the FIPV vaccine candidate. However, it remains imperative to conduct further *in vivo* investigations to validate the safety and efficacy of the identified epitopes and the proposed vaccine candidate. Future research endeavors could encompass the synthesis of the immunogenic epitopes identified herein, followed by an assessment of their capacity to trigger protective antibody responses in feline subjects. Furthermore, the proposed vaccine candidate might be cloned and expressed as a recombinant protein, facilitating its administration to FIPV-infected cats to gauge its immunogenicity and potential in conferring protection against FIPV infection.

Despite the potential of using immunoinformatics for designing FIPV multi-epitope vaccines, there are also some challenges. FIPV’s complexity, diverse strains, and mutants pose difficulties in accurately predicting epitopes. Bioinformatics tools may struggle to precisely forecast *in vivo* immunogenicity. Variability in individual immune responses, diverse MHC profiles, and an evolving understanding of host-virus interactions add further complexity. The accuracy of predicting T cell-stimulating epitopes through major histocompatibility complex (MHC) binding specificity varies, requiring frequent experimental validation. Effective presentation and processing of predicted epitopes by antigen-presenting cells may not be fully captured by bioinformatics. The uncertainty regarding the long-term durability of the immune response and the potential for FIPV to generate escape mutants pose challenges to vaccine success. Additionally, the regulatory approval process for vaccines developed using immunoinformatics may be hindered by the need for robust experimental validation and safety and efficacy concerns. Despite these limitations, combining computational and experimental approaches remains crucial for enhancing the effectiveness of FIPV multi-epitope vaccines.

## 5 Conclusion

The driving force of this investigation was the utilization of bioinformatics methodologies to pinpoint a prospective vaccine contender against FIPV, targeting the S-protein. An innovative vaccine candidate for FIPV was meticulously devised, boasting projected stability, non-allergenic characteristics, and anticipated antigenicity. Nevertheless, the trajectory ahead demands in-depth *in vivo* analyses to meticulously ascertain the safety and efficacy of the FIPV vaccine candidate. The suggested vaccine prospect emerges as an auspicious stepping stone, laying the groundwork for forthcoming experimental explorations that seek to cultivate an efficacious vaccine against FIPV.

## Data availability statement

The original contributions presented in the study are included in the article/Supplementary material, further inquiries can be directed to the corresponding authors.

## Author contributions

MC: Conceptualization, Methodology, Writing – original draft, Writing – review & editing, Data curation, Supervision, Validation, Visualization. AC: Data curation, Investigation, Writing – original draft. NA: Data curation, Methodology, Software, Writing – original draft. JM: Methodology, Software, Writing – original draft. SR: Data curation, Writing – original draft. CS: Data curation, Investigation, Writing – original draft. NJ: Data curation, Software, Writing – original draft. KC: Conceptualization, Funding acquisition, Writing – review & editing. AS: Formal analysis, Methodology, Software, Writing – review & editing. TM: Project administration, Writing – original draft. LC: Project administration, Resources, Supervision, Writing – review & editing.
