# Supplementary material for: Immunoinformatics-aided rational design of a multi-epitope vaccine targeting feline infectious peritonitis virus
Source: Front Vet Sci. 2023 Dec 13;10:1280273. doi: 10.3389/fvets.2023.1280273 (PMC10773687; doi:10.3389/fvets.2023.1280273)
Supplement: Supplementary file 1 [file Data_Sheet_1.docx]

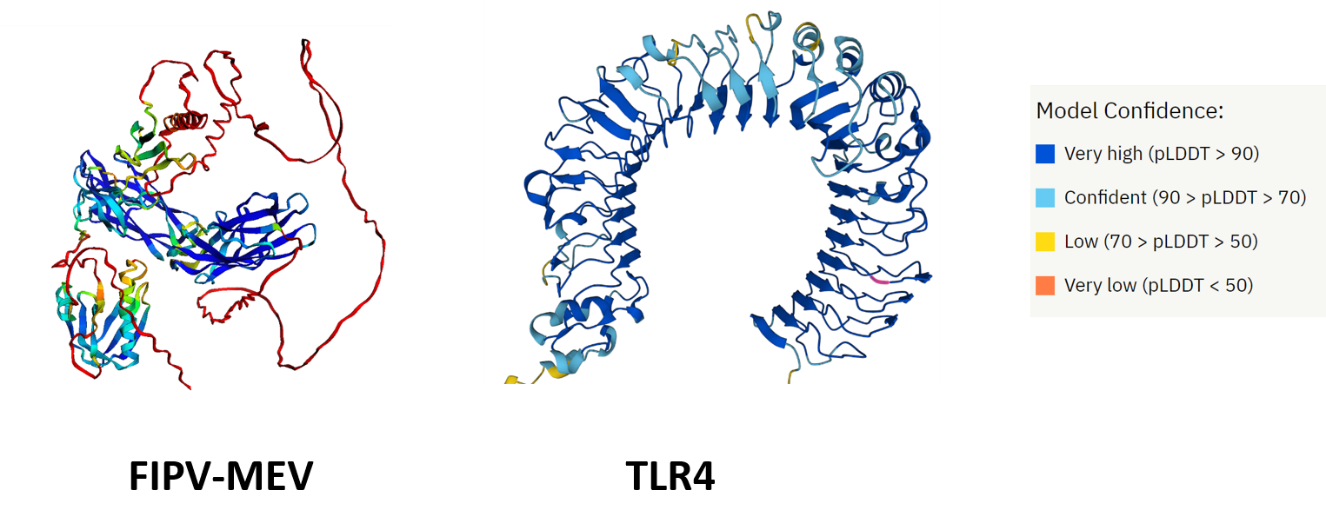


**Figure S1.** Modeled 3D structures of FIPV-MEV and TLR4 using AlphaFold and colored according to pLDDT scores.


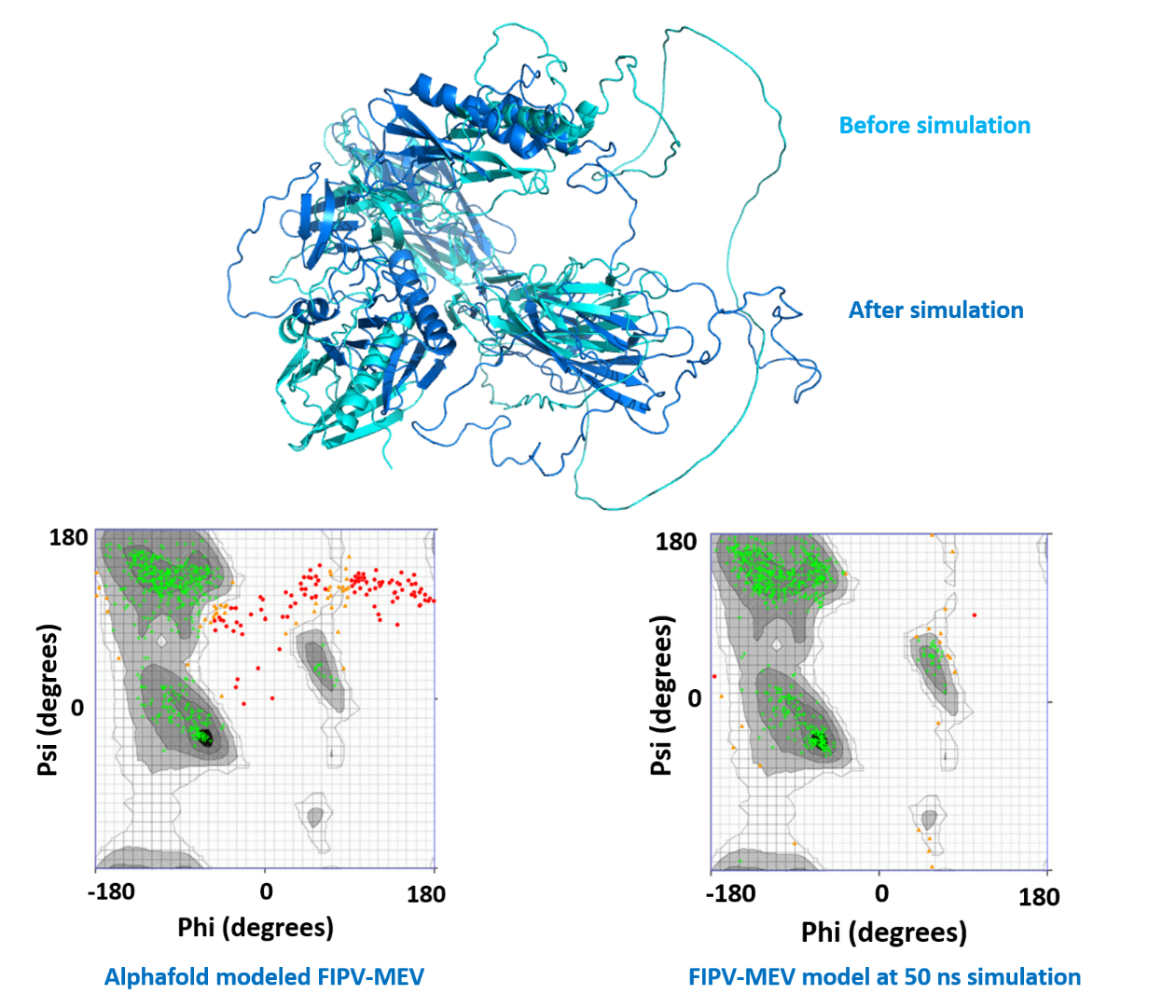


**Figure S2.** Superimposition of FIPV-MEV before and after simulation and their respective Ramachandran plots.

**Table S1**. Predicted epitopes for Tc cells identified as strong binders and selected for subsequent analysis

| **MHC** | **Peptide** | **Antigenicity-score-Vaxigen** | **Antigenicity** | **Allergenicity** | **Toxicity** |
| --- | --- | --- | --- | --- | --- |
| -------------- | ------------- |  |  |  |  |
| DLA-8803401 | VVKDVQLTL | -1.2609 | Antigen | Allergen | Non-Toxic |
| DLA-8803401 | KLHNTTVEL | 0.7915 | Antigen | Allergen | Non-Toxic |
| DLA-8803401 | VLLPSFFTY | -0.0127 | Non-Antigen | Allergen | Non-Toxic |
| DLA-8803401 | YAYQGVSNF | 0.5752 | Antigen | Non-Allergen | Non-Toxic |
| DLA-8803401 | FSFDKLNNY | 0.5554 | Antigen | Allergen | Non-Toxic |
| DLA-8803401 | SISDIYNRL | 0.5769 | Antigen | Allergen | Non-Toxic |
| DLA-8803401 | AISKWGHFY | -0.1998 | Non-Antigen | Allergen | Non-Toxic |
| DLA-8803401 | ALSHLTVQL | 0.8919 | Antigen | Non-Allergen | Non-Toxic |
| DLA-8803401 | YISGRSYHL | 0.8704 | Antigen | Non-Allergen | Non-Toxic |
| DLA-8803401 | FTISVQVEY | 1.4045 | Antigen | Allergen | Non-Toxic |
| DLA-8803401 | FVNKSVVLL | 0.678 | Antigen | Allergen | Non-Toxic |
| DLA-8803401 | ITKNRHINY | 1.3569 | Antigen | Non-Allergen | Non-Toxic |
| DLA-8803401 | ATWEYSAAY | 0.4778 | Antigen | Non-Allergen | Non-Toxic |
| DLA-8803401 | CVRSQSQRF | -0.2227 | Non-Antigen | Allergen | Non-Toxic |
| DLA-8803401 | AIGNITQAF | 0.5342 | Antigen | Allergen | Non-Toxic |
| DLA-8803401 | NARGKPLLF | 1.1829 | Antigen | Non-Allergen | Non-Toxic |
| DLA-8803401 | IIRRTNSTL | 0.5367 | Antigen | Allergen | Non-Toxic |
| DLA-8803401 | QVYTTPVSI | 0.6554 | Antigen | Allergen | Non-Toxic |
| DLA-8803401 | TVQDILENY | -0.1865 | Non-Antigen | Allergen | Non-Toxic |
| DLA-8803401 | KFYLTPRTM | 1.112 | Antigen | Allergen | Non-Toxic |
| DLA-8803401 | FVSENALKL | 0.0992 | Non-Antigen | Allergen | Non-Toxic |
| DLA-8803401 | FLLTNSSTF | 0.0591 | Non-Antigen | Non-Allergen | Non-Toxic |
| DLA-8803401 | SVKEIAISK | 0.5811 | Antigen | Allergen | Non-Toxic |
| DLA-8803401 | VLNKNQQIL | 0.54433 | Antigen | Allergen | Non-Toxic |
| DLA-8803401 | ITIDLGMKL | 1.6088 | Antigen | Allergen | Non-Toxic |
|  |  |  |  |  |  |
|  |  |  |  |  |  |
| DLA-8850101 | SISDIYNRL | 0.5769 | Antigen | Allergen | Non-Toxic |
| DLA-8850101 | FVNKSVVLL | 0.678 | Antigen | Allergen | Non-Toxic |
| DLA-8850101 | YISGRSYHL | 0.8704 | Antigen | Non-Allergen | Non-Toxic |
| DLA-8850101 | VVKDVQLTL | 1.2609 | Antigen | Allergen | Non-Toxic |
| DLA-8850101 | FVSENALKL | 0.0992 | Non-Antigen | Allergen | Non-Toxic |
| DLA-8850101 | SINSELLGL | 0.6903 | Antigen | Non-Allergen | Non-Toxic |
| DLA-8850101 | ALSHLTVQL | 0.8919 | Antigen | Non-Allergen | Non-Toxic |
| DLA-8850101 | TVAKALAKV | -0.0315 | Non-Antigen | Allergen | Non-Toxic |
| DLA-8850101 | MIFFHTVLL | 0.2256 | Non-Antigen | Allergen | Non-Toxic |
| DLA-8850101 | NINNTLVNL | 0.8715 | Antigen | Allergen | Non-Toxic |
| DLA-8850101 | FTADVQSGM | 0.7548 | Antigen | Allergen | Non-Toxic |
| DLA-8850101 | FVNATVIDL | 1.0835 | Antigen | Allergen | Non-Toxic |
| DLA-8850101 | VVNTQGQAL | 0.3175 | Non-Antigen | Allergen | Non-Toxic |
| DLA-8850101 | AIHQTSQGL | 0.7751 | Antigen | Non-Allergen | Non-Toxic |
| DLA-8850101 | ITIDLGMKL | 1.6088 | Antigen | Allergen | Non-Toxic |
| DLA-8850101 | HVHGEPVSV | 0.598 | Antigen | Allergen | Non-Toxic |
| DLA-8850101 | LITGRLTAL | 0.9134 | Antigen | Non-Allergen | Non-Toxic |
| DLA-8850101 | QVYTTPVSI | 0.6554 | Antigen | Allergen | Non-Toxic |
| DLA-8850101 | KLHNTTVEL | 0.7915 | Antigen | Allergen | Non-Toxic |
| DLA-8850101 | TISVQVEYM | 1.2198 | Antigen | Allergen | Non-Toxic |
| DLA-8850101 | YVALQTDVL | 0.0126 | Non-Antigen | Non-Allergen | Non-Toxic |
| DLA-8850101 | AIVGAMTSI | 0.488 | Antigen | Allergen | Non-Toxic |
| DLA-8850101 | LIDNINNTL | 0.1831 | Non-Antigen | Allergen | Non-Toxic |
| DLA-8850101 | TVQDILENY | -0.1865 | Non-Antigen | Allergen | Non-Toxic |
| DLA-8850101 | GVSGAFWTI | 0.1079 | Non-Antigen | Non-Allergen | Non-Toxic |
| DLA-8850101 | SVQVEYMQV | 1.0246 | Antigen | Allergen | Non-Toxic |
| DLA-8850101 | AMTSINSEL | 0.5706 | Antigen | Allergen | Non-Toxic |
| DLA-8850101 | YAYQGVSNF | 0.5752 | Antigen | Non-Allergen | Non-Toxic |
| DLA-8850101 | TVIDLPSII | 0.2816 | Non-Antigen | Allergen | Non-Toxic |
| DLA-8850101 | VIYEEGDNI | -0.2274 | Non-Antigen | Allergen | Non-Toxic |
| DLA-8850101 | SIIPDYIDI | 0.9515 | Antigen | Allergen | Non-Toxic |
| DLA-8850101 | AVQARLNYV | 1.3016 | Antigen | Allergen | Non-Toxic |
| DLA-8850101 | YLGTLPPSV | 0.4487 | Antigen | Allergen | Non-Toxic |
| DLA-8850101 | TVDVIRFNL | -0.0128 | Non-Antigen | Non-Allergen | Non-Toxic |
| DLA-8850101 | KVNDAIHQT | 0.5043 | Antigen | Allergen | Non-Toxic |
| DLA-8850101 | YVLYNGTAL | 0.2749 | Non-Antigen | Allergen | Non-Toxic |
| DLA-8850101 | VAIPFAVAV | 0.873 | Antigen | Non-Allergen | Non-Toxic |
| DLA-8850101 | FAVAVQARL | 1.1472 | Antigen | Non-Allergen | Non-Toxic |
|  |  |  |  |  |  |
| DLA-8850801 | SIYNYTSER | -0.0421 | Non-Antigen | Allergen | Non-Toxic |
| DLA-8850801 | FSFDKLNNY | 0.5554 | Antigen | Allergen | Non-Toxic |
| DLA-8850801 | SISDIYNRL | 0.5769 | Antigen | Allergen | Non-Toxic |
| DLA-8850801 | SVKEIAISK | 0.5811 | Antigen | Allergen | Non-Toxic |
| DLA-8850801 | RQAEVRASR | 0.1026 | Non-Antigen | Non-Allergen | Non-Toxic |
| DLA-8850801 | VLLPSFFTY | -0.0127 | Non-Antigen | Allergen | Non-Toxic |
| DLA-8850801 | FTISVQVEY | 1.4045 | Antigen | Allergen | Non-Toxic |
| DLA-8850801 | TVQDILENY | -0.1865 | Non-Antigen | Allergen | Non-Toxic |
| DLA-8850801 | GVSNFTYYK | 0.367 | Non-Antigen | Non-Allergen | Non-Toxic |
| DLA-8850801 | VLYNGTALK | 0.1488 | Non-Antigen | Non-Allergen | Non-Toxic |
| DLA-8850801 | VVKDVQLTL | 1.2609 | Antigen | Allergen | Non-Toxic |
| DLA-8850801 | FVNKSVVLL | 0.678 | Antigen | Allergen | Non-Toxic |
| DLA-8850801 | ALSHLTVQL | 0.8919 | Antigen | Non-Allergen | Non-Toxic |
| DLA-8850801 | SSISDIYNR | 0.7205 | Antigen | Allergen | Non-Toxic |
| DLA-8850801 | KLHNTTVEL | 0.7915 | Antigen | Allergen | Non-Toxic |
| DLA-8850801 | TTTPNFYYY | 0.7548 | Antigen | Allergen | Non-Toxic |
| DLA-8850801 | YAYQGVSNF | 0.5752 | Antigen | Non-Allergen | Non-Toxic |
| DLA-8850801 | KVNDAIHQT | 0.5043 | Antigen | Allergen | Non-Toxic |
| DLA-8850801 | FVSENALKL | 0.0992 | Non-Antigen | Allergen | Non-Toxic |
| DLA-8850801 | ATWEYSAAY | 0.4778 | Antigen | Non-Allergen | Non-Toxic |
| DLA-8850801 | YISGRSYHL | 0.8704 | Antigen | Non-Allergen | Non-Toxic |
| DLA-8850801 | VQVEYMQVY | 0.6392 | Antigen | Allergen | Non-Toxic |
| DLA-8850801 | AISKWGHFY | -0.1998 | Non-Antigen | Allergen | Non-Toxic |
| DLA-8850801 | FTADVQSGM | 0.7548 | Antigen | Allergen | Non-Toxic |
| DLA-8850801 | ITIDLGMKL | 1.6088 | Antigen | Allergen | Non-Toxic |
| DLA-8850801 | SVYVHSTCK | 0.328 | Non-Antigen | Allergen | Non-Toxic |
